# The miR166–mRNA network regulates vascular tissue differentiation in Moso bamboo

**DOI:** 10.3389/fgene.2022.893956

**Published:** 2022-08-12

**Authors:** Ying Li, Shuqin Zhang, Deqiang Zhang, Xueping Li, Zhimin Gao, Zehui Jiang

**Affiliations:** ^1^ National State Forestry and Grassland Administration Key Open Laboratory on the Science and Technology of Bamboo and Rattan, Institute of Gene Science and Industrialization for Bamboo and Rattan Resources, International Centre for Bamboo and Rattan, Beijing, China; ^2^ National Engineering Laboratory for Tree Breeding, College of Biological Sciences and Technology, Beijing Forestry University, Beijing, China

**Keywords:** miR166, vascular tissue, xylem, differentiation, transcription factor, bamboo, seedling

## Abstract

miR166s play an important role in plant tissue differentiation. However, the functions of miR166s in the differentiation of vascular tissue in bamboo have not yet been elucidated. Here, we showed that five miR166s are overexpressed (tags per million reads > 2,000) in underground shoot samples of wild-type (WT) Moso bamboo (*Phyllostachys edulis*) and a thick-walled variant (*P. edulis* “Pachyloen”) throughout the developmental process. Potential targets of these miR166s include some genes encoding homeodomain-leucine zipper (HD-ZIP) transcription factors and protein kinases. Cleavage sites for miR166s were identified in seven *PeHD-ZIP* homologs and a protein kinase gene *via* degradome sequencing (*p* < 0.05). Dual-luciferase and transient expression assays confirmed the binding of miR166s to *PeHOXs*. Fluorescence *in situ* hybridization revealed that miR166s were localized to the xylem of the leaf, root, and internode of 2-month-old pot seedlings of WT Moso bamboo. Overall, these findings reveal that miR166s are regulators of vascular tissue differentiation in bamboo. The miR166s identified in our study provide novel targets for bamboo breeding.

## Introduction

**FIGURE 1:**
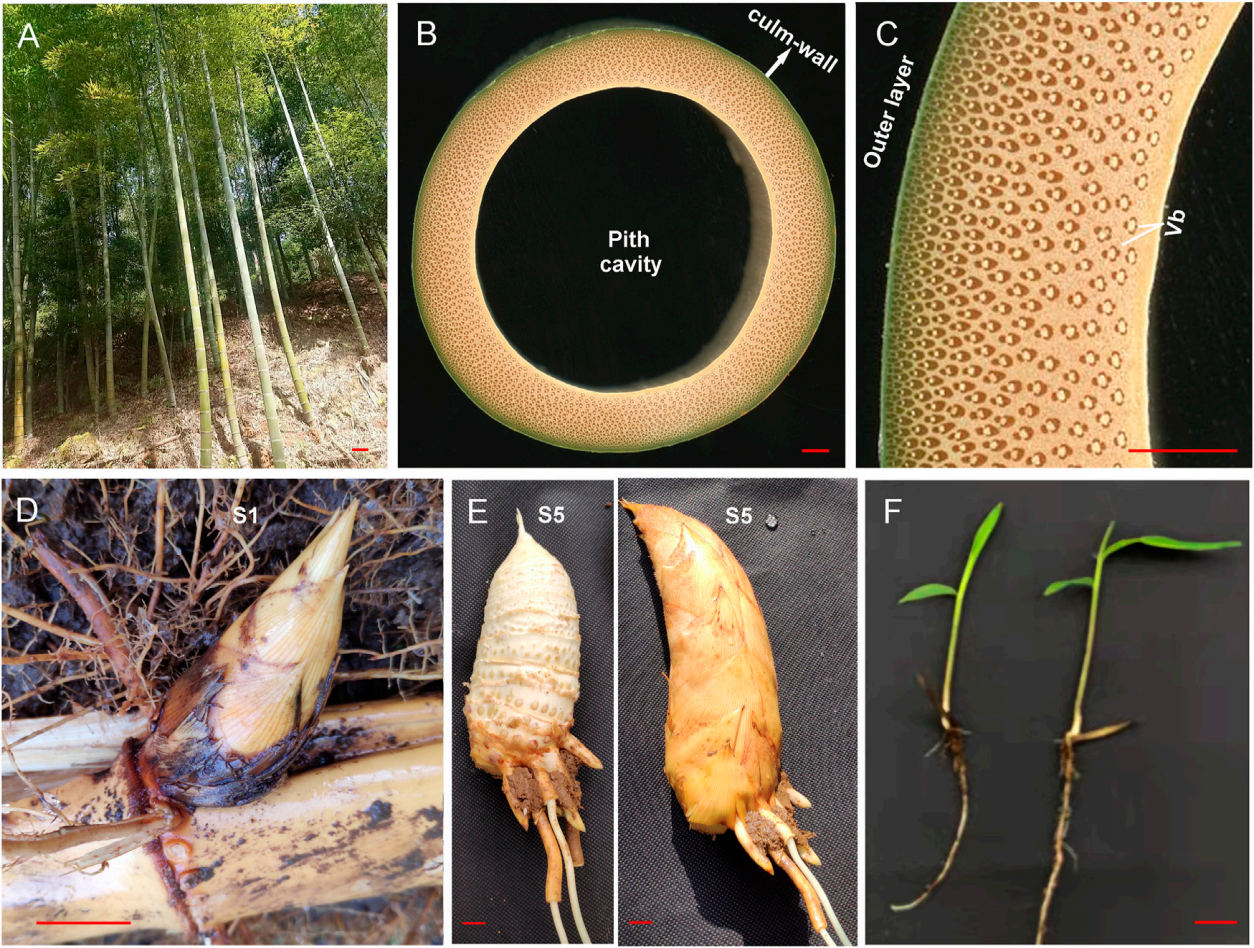
Phenotypes of aboveground culms, underground shoots, and seedlings of Moso bamboo. **(A)** Aboveground culms of two-year-old wild-type Moso bamboo plants. **(B)** Cross-sections of the third internode of the mature culm of wild-type Moso bamboo. **(C)** Enlarged image of the local culm wall in **(B)**. **(D)** Underground shoots collected at the S1 stage. **(E)** Underground shoots collected at the S5 stage; the left image shows the edible bamboo shoot with the shell removed. **(F)** Phenotype of two-month-old Moso bamboo seedlings. The scale bar is 10 cm in **(A)** and 1 cm in the other panels. Vb, vascular bundle.

Moso bamboo (*Phyllostachys edulis* (Carr.) H. de Lehaie) is a member of the subfamily Bambusoideae in the family Poaceae. Its ability to grow rapidly, coupled with the high quality of the wood of its culms, distinguishes it from rice (*Oryza sativa* L.), other herbaceous plants in the family Poaceae, and dicotyledonous woody and fruit trees. The culm wall of bamboo has a graded hierarchical structure, and vascular bundles (VBs) provide support for this structure (Wei et al., 2019) (Figures 1A–C). The tender shoots of bamboo, which are located at the nodes of well-developed rhizomes (Figures 1D,E), possess a unique taste and nutritional profile due to VBs. VBs provide support to the culm, which is more than 10 m tall; they form a dense network for the large underground rhizomatous system and confer the unique taste and nutrient profile of the shoots. However, the molecular mechanism underlying vascular tissue differentiation remains unclear. The aboveground part of bamboo seedlings is similar to that of rice; however, VBs are densely distributed in parenchyma cells of the internode, and regular changes in the radial direction of the culm wall are often observed, which is in contrast to the morphology of the vascular tissue in rice. Additional studies are needed to elucidate the molecular mechanisms underlying the regulation of the vascular tissues, including the differentiation of vascular tissues in the shoots and seedlings, as such studies could shed light on how variation in regulatory mechanisms drives the formation of specific phenotypes. There is a special need for studies aimed at identifying candidate regulatory elements that affect bamboo biomass and the properties of wood.

In plants, miR166/165 members comprise an important group of microRNAs (miRNAs) that play key roles in meristem development and organ polarity (Byrne, 2006; Zhou et al., 2007) by regulating the expression of various target genes, such as class III homeodomain/leucine zipper (*HD-ZIP III*) transcription factor (TF) genes (Prigge et al., 2005). Argonaute 10 inhibits the activity of miR165/166, which leads to the localized enrichment of *HD-ZIP* transcripts, ensures that the shoot apical meristem is correctly programmed in developing embryos, and supports the formation of the adaxial domain, the vasculature of the cotyledons, and the new leaf primordia (Zhang and Zhang, 2012; Zhou et al., 2015). In rice, miR166 mediates the localization of the RDD1 protein in VBs and sheaths by repressing the expression of the TF gene *rice Dof daily fluctuations 1* (*RDD1*) in the mesophyll (Iwamoto and Tagiri, 2016). During root procambial development in *Arabidopsis*, a network composed of miR166/165, *PHLOEM EARLY DOF 1* and *2* (*PEAR1* and *2*), and the four homologs *DOF6*, *TM O 6*, *OBP2*, and *HCA2*, integrates spatial information of the hormonal domains and miRNA gradients to generate adjacent zones of dividing and quiescent cells, which facilitates radial growth (Miyashima et al., 2019). miRNAs have also been identified in the aboveground parts of mature bamboo culms (Ge et al., 2017). miRNAs have been shown to play an important role in the tissue differentiation of underground shoots at stages S1–S5 in wild-type (WT) Moso bamboo and its thick-walled (TW) variant (*P. edulis* “Pachyloen”), and miR166s are located in the vascular tissues of Moso bamboo shoots (Li et al., 2022). However, the roles of miR166s in the aforementioned regulatory network remain unclear; furthermore, little is known about their function in vascular tissue differentiation in bamboo seedlings.

Here, we aimed to 1) identify the target genes of miR166s, 2) study the interplay between miR166s and their targets, 3) characterize the localization of miR166s in bamboo shoots and seedlings, and 4) evaluate the implications of localization differences for tissue differentiation. We performed an integrated analysis of miR166s with the transcriptome, miRNAome, and degradome sequencing data using underground shoot samples of the WT Moso bamboo and the TW variant *P. edulis* “Pachyloen” taken throughout five stages of development (WTS1/TWS1–WTS5/TWS5) (Li et al., 2022). We also performed dual-luciferase reporter assays to study the interplay between miR166s and their predicted targets and RNA fluorescence *in situ* hybridization (FISH) to determine the localization of miR166s in leaf, root, and internode samples collected from two-month-old bamboo seedlings (Figure 1F). The results of our study provide new insights into the role of miR166s in vascular tissue differentiation and new candidate regulatory elements that could be used to enhance the biomass and wood properties of Moso bamboo.

## Materials and methods

### Plant materials

To determine the localization of miR166s, we collected two-month-old seedlings of Moso bamboo that had been maintained in an air-conditioned greenhouse; they were located in the National State Forestry and Grassland Administration Key Open Laboratory of Science and Technology of Bamboo and Rattan, Beijing, China (39˚59′ 17.52″ N, 116̊ 28′46.06″ E). Plants were watered three times a week and maintained at 25 ± 1°C and 55 ± 5% relative humidity under a 16/8 h (light/dark) photoperiod with a light intensity of 1,250 μmol m^−2^ s^−1^.

### Data collection

The transcriptome, small RNA, and degradome sequencing data of underground shoot samples of WT and the TW Moso bamboo at the WTS1/TWS1-WTS5/TWS5 stages were downloaded from the NCBI SRA database using the accession No. PRJNA753616 (https://www.ncbi.nlm.nih.gov/Traces/study/?acc=PRJNA753616) (Li et al., 2022).

### Identification and annotation of miR166s

The downloaded small RNA sequencing reads were screened against various databases, such as Rfam (http://rfam.xfam.org/), Silva (http://www.arb-silva.de/), GtRNAdb (http://lowelab.ucsc.edu/GtRNAdb/), and Repbase (http://www.girinst.org/repbase/), containing data on ribosomal RNAs (rRNAs), transfer RNAs (tRNAs), small nuclear RNAs, small nucleolar RNAs (snoRNAs), and other noncoding RNAs as well as repeat sequences using Bowtie v1.0.0 (Langmead et al., 2009) with the parameters “-v 0”. To identify known miRNAs, the unannotated reads were mapped to the Moso bamboo reference genome v2.0 (Zhao et al., 2018), and the successfully mapped reads were compared against miRNAs of close relatives of Moso bamboo (*Brachypodium distachyon* and *Oryza sativa*) in miRBase (Kozomara et al., 2019) (release 22, http://mirbase.org/) using Bowtie v1.0.0., as miRNA annotations to the Moso bamboo genome and Moso bamboo miRNAs in miRbase were lacking (Zhao et al., 2018). Precursor sequences were mapped to the reference Moso bamboo genome using Bowtie v1.0.0.

Unaligned unique reads were used for the prediction of novel miRNAs in miRDeep-P2 (Kuang et al., 2019) following newly updated criteria for plant miRNA annotations (Axtell and Meyers, 2018) as well as the method described by Guo et al. (2020). miRNAs identified with similar sequences were clustered into families.

### Expression analysis of miR166s

The abundances of miR166s were normalized to tags per million (TPM) (Li et al., 2010) using the following equation:

$$\text{TPM}=\frac{\text{Readcount}*1,000,000}{\text{Mapped}\;\text{Reads}},$$

where Readcount and Mapped Reads represent the number of reads mapped onto certain miRNAs and those mapped onto all miRNAs in the reference genome v2.0, respectively.

Replicates with high consistency (*r* ^2^ > 0.70) according to the Pearson’s correlation analysis of gene expression (Li et al., 2022) were used in subsequent analyses.

We identified differentially expressed miRNAs (DEmiRs) between two stages for 13 pairwise comparisons of the WT, TW, and WTTW groups using DESeq2 (Love et al., 2014), including TWS2_vs._WTS1/TWS1, WTS3/TWS3_vs._WTS2/TWS2, WTS4/TWS4_vs._WTS3/TWS3, and WTS5/TWS5_vs._WTS4/TWS4 for the WT/TW group and TWS1–5_vs._WTS1–5 for the WTTW group. miRNAs were considered differentially expressed according to the following criteria: TPM > 1, |log_2_(FC)| ≥ 1.00, and false discovery rate (FDR)-corrected *p*-value < 0.05 in one of two pairwise comparisons. Comparisons of derived DEmiRs were referred to as “A_vs._B”, for example, S01_vs._S02 corresponds to the comparison of DEmiRs between S01 and S02.

### Target prediction and annotation

Potential miR166 targets were identified using psRNATarget (Dai et al., 2018) and the default parameters of Schema V2 (2017 release) (Dai et al., 2018), with the only difference being that a threshold value of 3 was used.

Annotations of these sequences were performed by conducting queries against several databases, including the Pfam (http://pfam.xfam.org/), Swiss-Prot (http://www.uniprot.org/), Clusters of Orthologous Genes (http://www.ncbi.nlm.nih.gov/COG/), Eukaryotic Orthologous Groups (http://www.ncbi.nlm.nih.gov/KOG/), Gene Ontology (http://www.geneontology.org/), Kyoto Encyclopedia of Genes and Genomes (http://www.genome.jp/kegg/), and NR (ftp://ftp.ncbi.nih.gov/blast/db/) databases, using BLAST v2.2.26 (Altschul et al., 1997).

### Expression analysis of predicted target genes

Downloaded transcriptome sequencing reads were mapped to the Moso bamboo reference genome v2.0 using HISAT2 v2.0.4 (Kim et al., 2015) with the parameters “--dta -p 6 --max-intronlen 5000000” to identify known genes. The reads mapped to the unannotated regions were then assembled using StringTie v1.3.4d (Pertea et al., 2015) with the parameters “--merge -F 0.1 -T 0.1” to identify novel genes.

Gene expression levels were estimated using fragments per kilobase of transcript per million fragments mapped (FPKM) (Florea et al., 2013) using the following formula:

$$\text{FPKM}=\frac{\text{cDNA}\;\text{Fragments}}{\text{Mapped}\;\text{Fragments }\left(\text{Millions}\right)\;\times \text{ Transcript}\;\text{Length }\left(\text{kb}\right)}.$$

Replicates with high consistency (*r* ^2^ > 0.70) according to the `Pearson’s correlation analysis (Li et al., 2022) were used in subsequent analyses.

DESeq2 was used to identify DEGs (Love et al., 2014). DEGs were identified using the following criteria: FPKM > 1, |log_2_(FC)| ≥ 1.00, and FDR-corrected *p*-value < 0.01 in one of two pairwise comparisons. Comparisons of derived DEGs were referred to as “A_vs._B”, for example, WT1_vs._WT2 corresponds to DEGs between WTS1 and WTS2.

### Degradome analysis of miR166–mRNA pairs

Downloaded degradome sequencing reads were searched against all other noncoding RNA sequences from Rfam, with the exception of miRNAs, using Bowtie v1.0.0; reads aligned to rRNAs, tRNAs, snoRNAs, and repeats were removed. The clean reads obtained were mapped to the Moso bamboo reference genome (v2.0), and a maximum of one mismatch was allowed. The miRNA cleavage sites were predicted using the CleaveLand (v4.4) pipeline (Addo-Quaye et al., 2009); only category 0, 1, and 2 results were retained to reduce the incidence of false positives.

### Sequence analysis and phylogenetic tree construction

Gene structures of the predicted targets of miR166s were analyzed using bambooGDB (http://www.bamboogdb.org). Domains in the target protein sequences were detected using SMART (http://smart.embl-heidelberg.de). Phylogenetic trees were constructed using MEGA v5 (Tamura et al., 2011) with the neighbor-joining method and under the pairwise deletion and Poisson correction models. Bootstrapping was conducted using 1,000 replicates.

### Luciferase reporter assay

Mimics of miRNAs, mRNAs 200 bp in length, and mutant-type (MUT) mRNAs with site-directed mutations in the predicted splicing sites were artificially synthesized (Sangon Biotech, Shanghai, China). They were then cloned into the pmirGLO vector (GeneCreate, Wuhan, China) between *Nhe*I and *Xho*I (TaKaRa, Nojihigashi, Japan); the control group was transfected with empty plasmid.

After confirmation by sequencing, 293T cells were co-transfected with MUT and WT vectors containing negative control (NC) mimics and miRNA mimics (GeneCreate), respectively. Cells were collected and processed 48 h after transfection using the Luciferase Reporter Assay Kit (RG005, Beyotime, Shanghai, China) as per the manufacturer’s instructions. The outcomes were quantified in each well as the proportion of the firefly luciferase/*Renilla* luciferase activity. Three independent experiments were performed. The sequences of NC and miRNA mimics as well as those of WT and MUT plasmids, are listed in Supplementary Table S1-1.

### Transient expression assays in *Nicotiana benthamiana* leaves

#### DNA and RNA extraction

Total DNA was isolated from the leaves of two-month-old Moso bamboo seedlings using the DNeasy Plant Mini Kit (Tiangen, Beijing, China) per the manufacturer’s instructions. Total RNA was isolated from the roots, stems, and leaves of two-month-old Moso bamboo seedlings using the Tiangen RNAprep Pure Plant Kit (Tiangen) per the manufacturer’s instructions. After treatment with RNase-free DNase I (Tiangen), 1 μg of RNA was reverse-transcribed using TransScript^®^ II One-Step gDNA Removal and cDNA Synthesis SuperMix (TransGen Biotech).

#### Transient expression assay

Transient expression assays were performed following a previously described method (Wang et al., 2021) with slight modifications. Fragments of *PeHOX10* and *PeHOX32* with the predicted target sites were fused in the pCAMBIA1302 vector with the GFPuv sequence (Yuan et al., 2021) driven by the 35S promoter. The stem-loop sequence of ped-miR166a-3p was cloned and inserted into the pCAMBIA1300 vector after the 35S promoter. 35S::PeHOX10-uvGFP, 35S::PeHOX32-uvGFP, and 35S:ped-miR166a-3p constructs were transformed into *Agrobacterium*. These strains were co-infiltrated into the leaves of *N. benthamiana*. GFP signals were detected 48 h later under the UV light. Primers are shown in Supplementary Table S1-2.

### RNA fish

FISH assays were carried out to track the localization of miR166s in the cells and tissues of Moso bamboo seedlings. Paraffin-embedded tissues and sections were prepared following the method in Wei et al. (2017). The sections were first dewaxed in water, incubated with a mixed solution of 30% (w/v) H_2_O_2_ and methanol (1:10, v/v) at room temperature for 10 min, and then covered by proteinase K at 37°C for 20 min.

Hybridization was conducted using the FISH kit (C007, Shanghai Gefan Biotechnology Co., Ltd., Shanghai, China) as per the manufacturer’s protocol. Briefly, the tissues were washed with phosphate-buffered saline (PBS), fixed in 4% (w/v) paraformaldehyde, and acetylated in acetic anhydride, followed by incubation with fluorescein isothiocyanate (FITC)-labeled probes (Abiocenter, Beijing, China) overnight at 65°C. They were then washed with 2 × SSC buffer, formamide/4 × SSC (1:1, v/v), and PBS buffer. Cell nuclei were counterstained with 4,6-diamidino-2-phenylindole (Invitrogen), followed by washing with PBS and antifade buffer (Beyotime, Shanghai, China). Images were taken using a Nikon Eclipse Ci-L microscope with a FITC fluorescence filter. The probe sequence was as follows: 5′-TCG​GAC​CAG​GCT​TCA​TTC​CCC​C-3′.

### Statistical analyses

Statistical analyses were performed using SPSS 19.0 software (IBM, Armonk, NY, United States). All data in this study were presented as mean ± standard deviation, and all statistical tests were two-sided. Student’s *t*-tests were conducted to evaluate the significance of differences between groups, and the threshold for statistical significance was *p* < 0.05.

## Results

### A total of 487 predicted target genes of seven miR166s were detected

**FIGURE 2:**
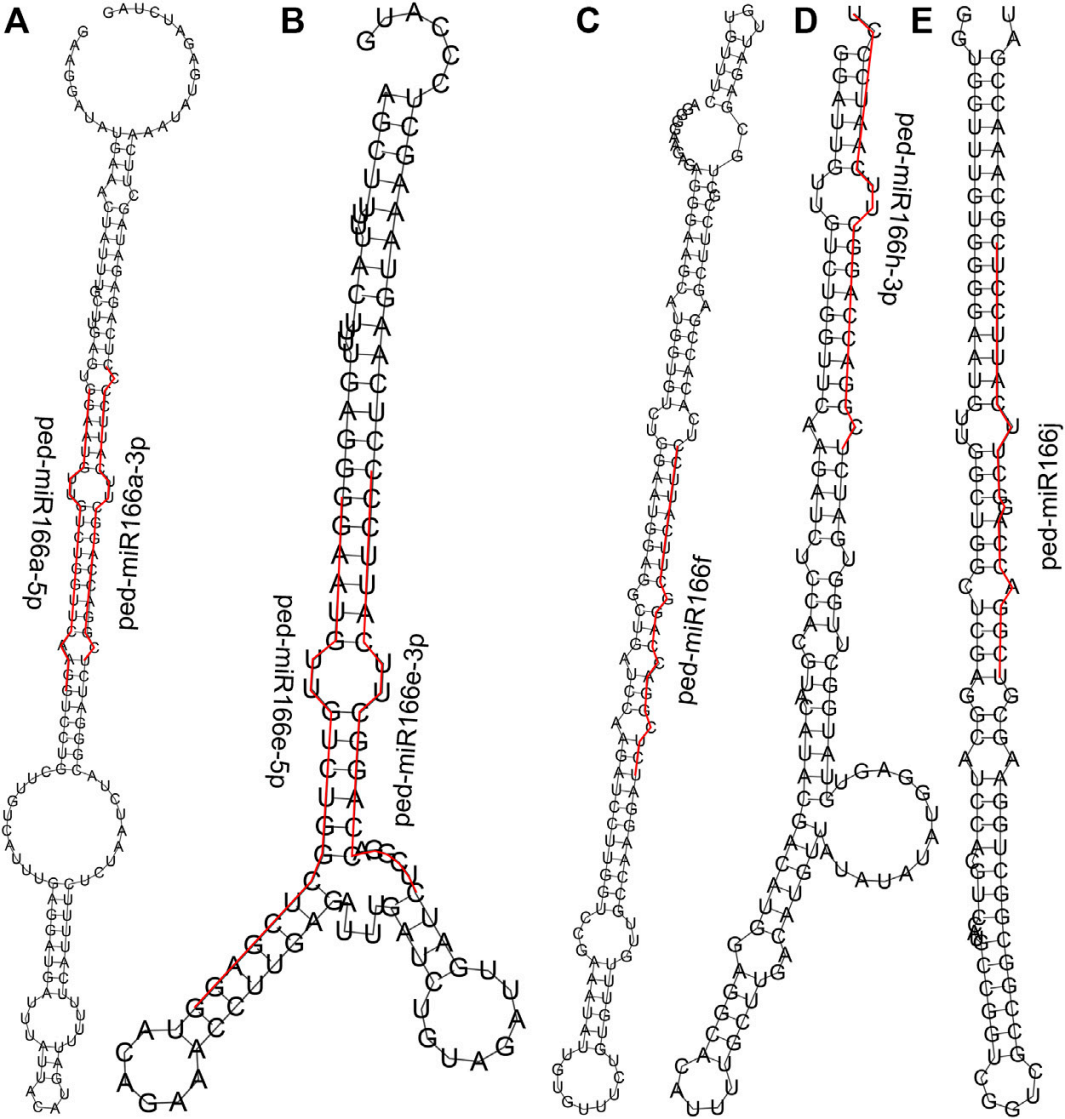
Predicted secondary structures of seven members of the miR166 family. Sequences of mature miR166s are denoted by the red line. Secondary structures predicted for ped-miR166a-3p and ped-miR166a-5p **(A)**, ped-miR166e-3p and ped-miR166e-5p **(B)**, ped-miR166f **(C)**, ped-miR166h-3p **(D)**, and ped-miR166j **(E)**.

We detected seven miR166s (Supplementary Table S2) according to the small RNA sequencing data from the underground shoots of WT and TW plants (Li et al., 2022). The length of all seven miR166s was 21 bp, and four (57.14%) had a 5′ uridine residue as the first base. The average GC content of miR166s was 57.14%. Their secondary structures varied (Figure 2), and the minimum free energy ranged from −68.30 kcal/mol (ped-miR166f) to −49.60 kcal/mol (ped-miR166h-3p) (Supplementary Table S2).

A total of 487 target genes were predicted for all seven miRNAs (Supplementary Table S2). Of these, 25 were TFs from 11 families. The most common TF families were HB-HD-ZIP (homeobox-homeodomain-leucine zipper; 10, 2.05%), NAC (NAM [no apical meristem], ATAF1/2 [*Arabidopsis* transcription activation factor1/2], and CUC2 [cup-shaped cotyledon2]; 5, 1.03%), and MYB-related (v-myb avian myeloblastosis viral oncogene homolog-related, 2, 0.41%) families. In addition, 100 and 16 genes encoding protein kinases (PKs) and transcriptional regulators (TRs) were identified, respectively.

### Five miR166s were overexpressed in underground bamboo shoots

All seven miR166s were expressed (TPM > 1) in at least one of the samples in WT and TW groups, and eight (88.89%) miRNAs were expressed in all five stages. Five and three miRNAs were overexpressed (TPM > 2,000) and highly overexpressed (TPM > 10,000) throughout all five stages in WT and TW plants, respectively (Supplementary Table S2).

A total of 423 miRNA-TF pairs comprised five overexpressed miR166s and 234 predicted targets (Supplementary Table S3), among which 25, 16, and 9 encoded TFs, PKs, and TRs, respectively. ped-miR166j was detected in most pairs (105), followed by ped-miR166f (98), ped-miR166e-3p (83), and ped-miR166h-3 (70).

**FIGURE 3:**
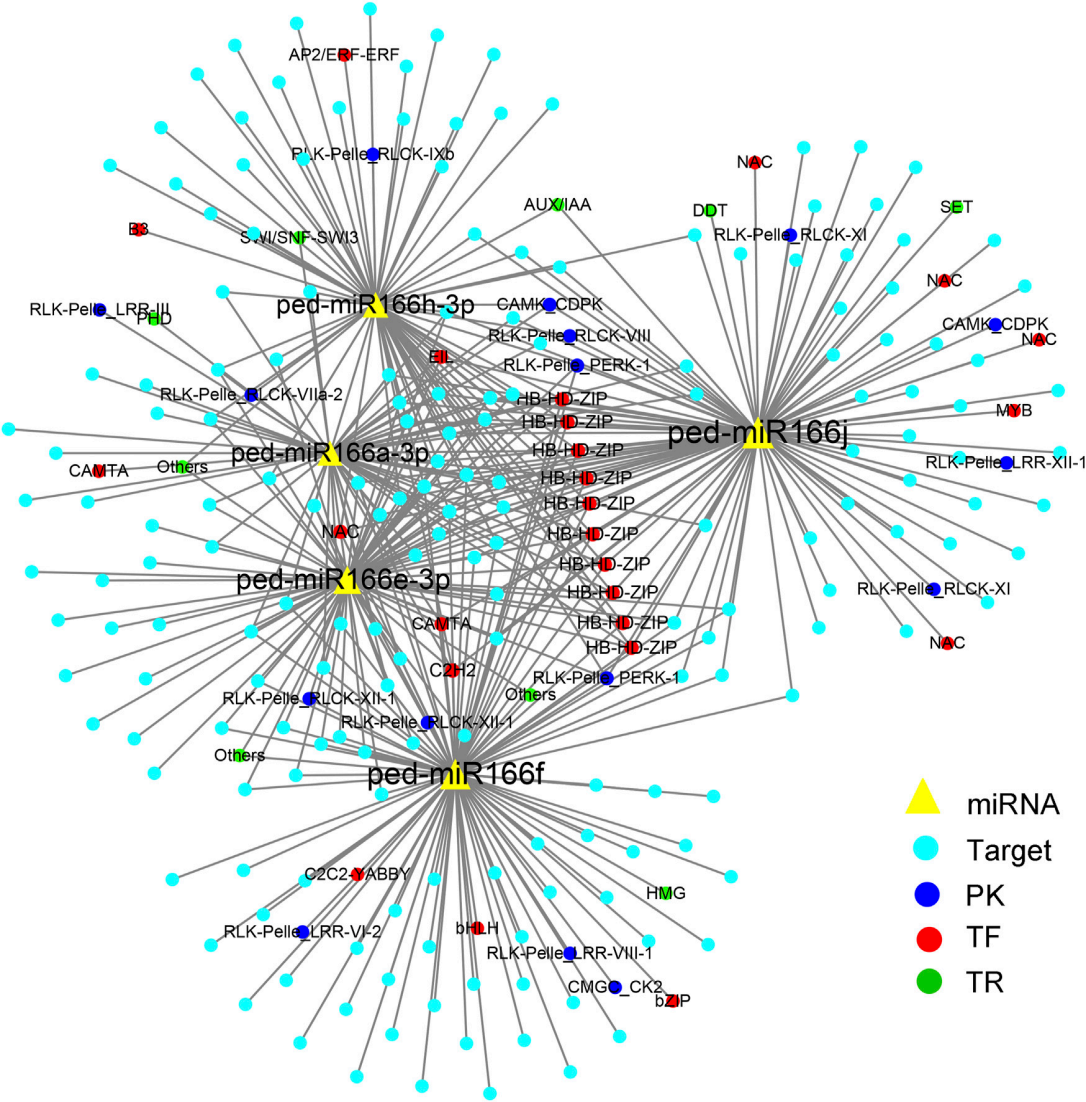
Cytoscape regulatory networks of five overexpressed miR166s and their predicted targets. Yellow triangles indicate miRNAs; blue solid circles indicate target genes. Targets that encode protein kinases are indicated by deep blue circles, transcription factors are indicated by red circles, and transcriptional regulators are indicated by green circles.

Nearly all five overexpressed miR166s shared targets from the HB-HD-ZIP TF family (Figure 3). The targets also included genes from other TF families, such as NAC, MYB, and AP2/ERF TFs. The most common PKs detected in miRNA–mRNA pairs were from the receptor-like kinase (RLK)/Pelle class (13).

### Two miR166s were differentially expressed in 13 pairwise comparisons

Only two (22.22%) miR166s, ped-miR166c-5p and ped-miR166h-3p, were differentially expressed (TPM > 1, FDR < 0.05, and |log_2_(FC)| ≥ 1 in one of two pairwise comparisons) among 13 pairwise comparisons (Supplementary Table S2). ped-miR166c-5p showed differential expression in the WTS5_vs._WTS4 comparison in the WT group and at S5 in the WTTW group. ped-miR166h-3p was differentially expressed in the WTS3_vs._WTS2 comparison in the WT group.

**FIGURE 4:**
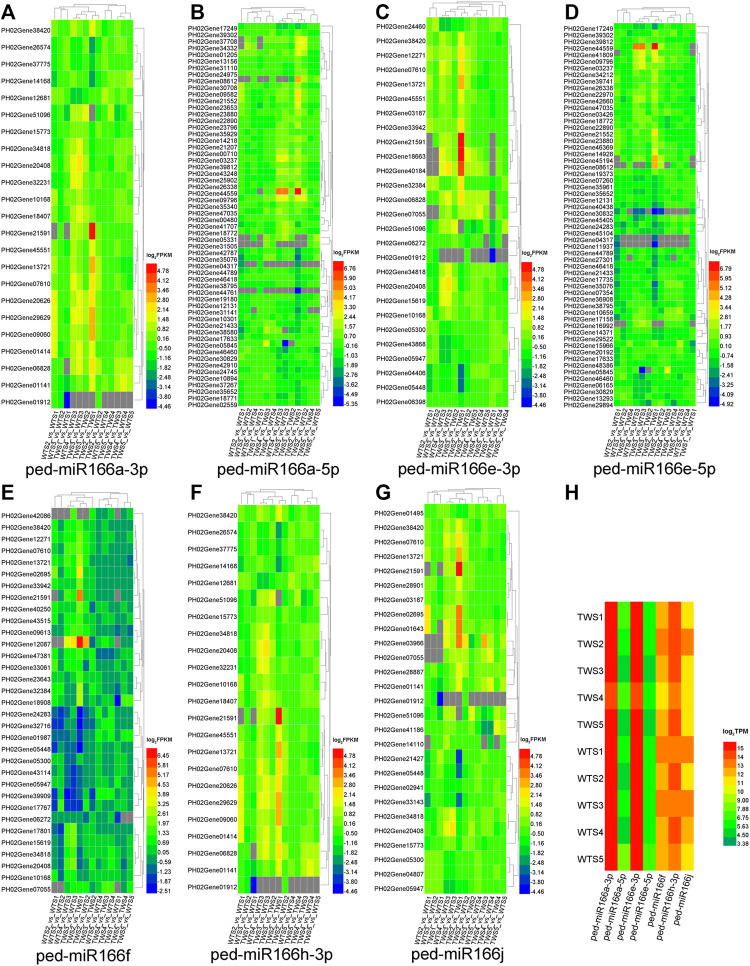
Expression patterns of seven miR166s and their target genes differentially expressed in 13 pairwise comparisons of the WT, TW, and WTTW groups. Both the wild-type (WT) and the thick-walled (TW) groups comprise four pairwise comparisons between two successive stages (WTS2/TWS2_vs_WTS1/TWS1, WTS3/TWS3_vs_WTS2/TWS2, WTS4/TWS4_vs_WTS3/TWS3, and WTS5/TWS5_vs_WTS4/TWS4), and the WTTW group comprises five pairwise comparisons between two stages (TWS1–5_vs_WTS1–5). **(A–G)** Expression patterns of the target genes of seven miR166s. **(H)** Expression patterns of seven miR166s. Gray squares indicate miRNAs and genes with low expression (TPM ≤ 1 or FPKM ≤ 1).

Target genes were identified for all seven members that were differentially expressed in at least one of the 13 comparison groups (Figure 4). Most of these genes were upregulated and/or downregulated in the TWS2_vs._TWS1 comparison. Among 235 targets of five overexpressed miR166s, 71 (30.21%) were differentially expressed (TPM > 1, FDR < 0.05, and |log_2_(FC)| ≥ 1 in one of the two pairwise comparisons) among 13 pairwise comparisons (Supplementary Table S3).

### A total of 23 miRNA–mRNA pairs were verified by degradome sequencing

**FIGURE 5:**
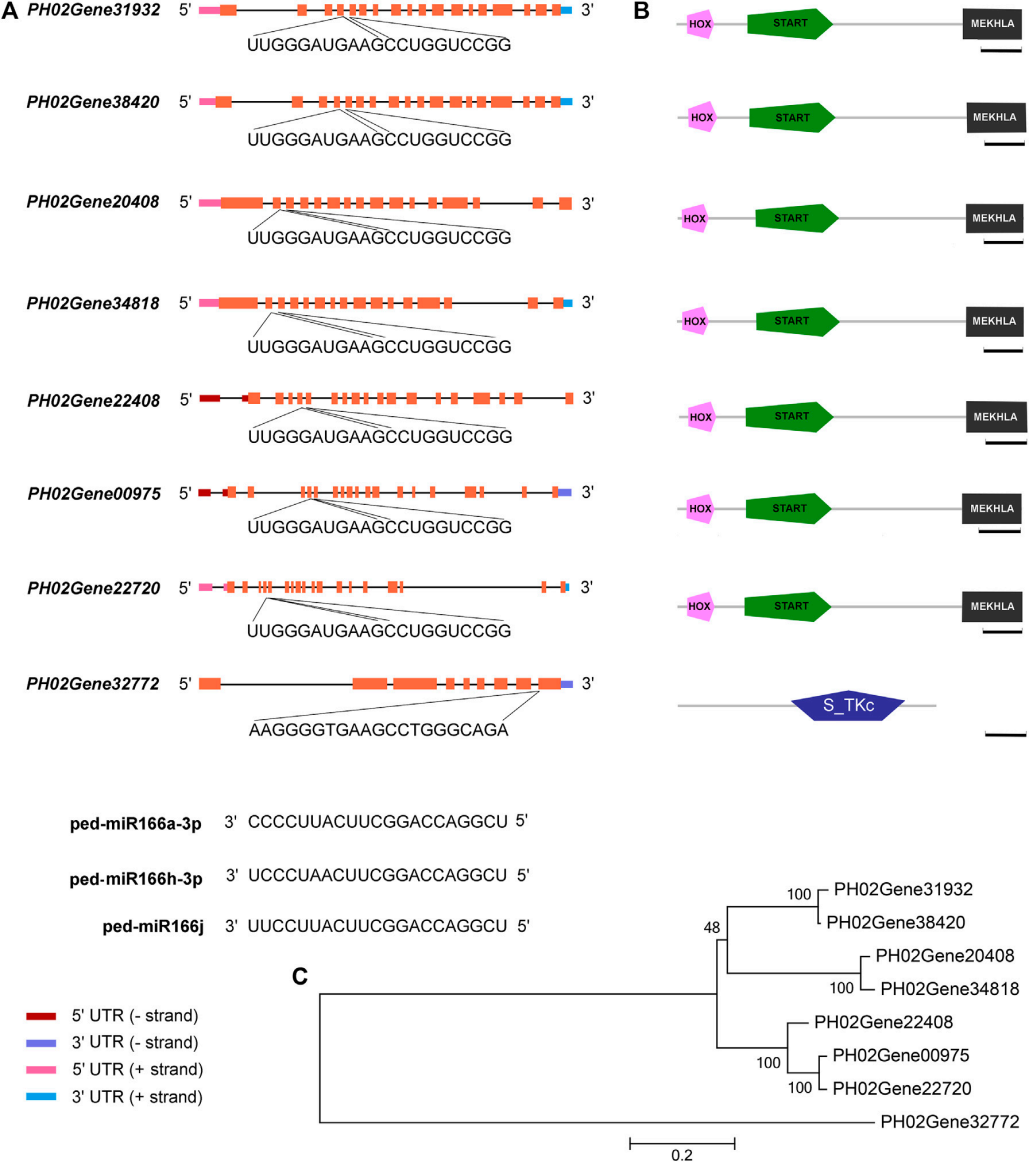
miR166s and their predicted targets in Moso bamboo. **(A)** Sequences of three miR166s, seven *HD-ZIP III* genes, and a protein kinase gene. **(B)** *HD-ZIP III* genes encode proteins with the following domains: an N-terminal MEKHLA domain, a START domain, and a C-terminal HOX domain. **(C)** Relationships among eight target genes. Diagrammatic representation of the target genes. Exons correspond to boxes, and introns correspond to lines. The HOX, START, and MEKHLA domains are the three structural domains in the coding sequences. Following them are the sequences of the binding sites in the eight targets and miR166s. Bar = 100 amino acids.

A total of seven cleavage sites were identified for 23 miRNA-mRNA pairs, which included three miR166s and eight targets, and eight cleavage sites were identified (*p* < 0.05) in WT and TW groups (Figure 5A, Supplementary Table S4). Seven targets had HOX, START, and MEKHLA domains, and they were clustered into a single group (Figures 5B,C). HOX is a DNA-binding factor involved in the transcriptional regulation of key developmental processes, and START (StAR-related lipid-transfer) is a lipid-binding domain in the StAR and HD-ZIP proteins as well as other signaling proteins. The MEKHLA domain is present in the 3′ end of plant HD-ZIP III homeobox genes, which encode DNA-binding factors involved in the transcriptional regulation of key developmental processes. The S_TKc domain is present in the serine or threonine-specific kinase subfamily of phosphotransferases.

Several miR166s within the same family cleave the same target. For example, *PH02Gene00975*, which encodes a putative *PeHOX10* homolog of rice, had the same cleavage site for ped-miR166a-3p, ped-miR166h-3p, and ped-miR166j in both WT and TW groups (Supplementary Table S4-1). The number of miR166–mRNA pairs differed among groups (Supplementary Table S4-2,3). A total of 21 and 17 cleavage sites were identified in the WT and TW groups, respectively. *PH02Gene20408* and *PH02Gene34818* had the same cleavage site for ped-miR166h-3p and ped-miR166j in the WT group, whereas no cleavage site was detected for these miRNAs in the TW group. *PH02Gene32772* had the same cleavage site for ped-miR166h-3p and ped-miR166j only in the TW group.

### miR166s specifically bound to the *PeHOX* transcriptional factor genes

**FIGURE 6:**
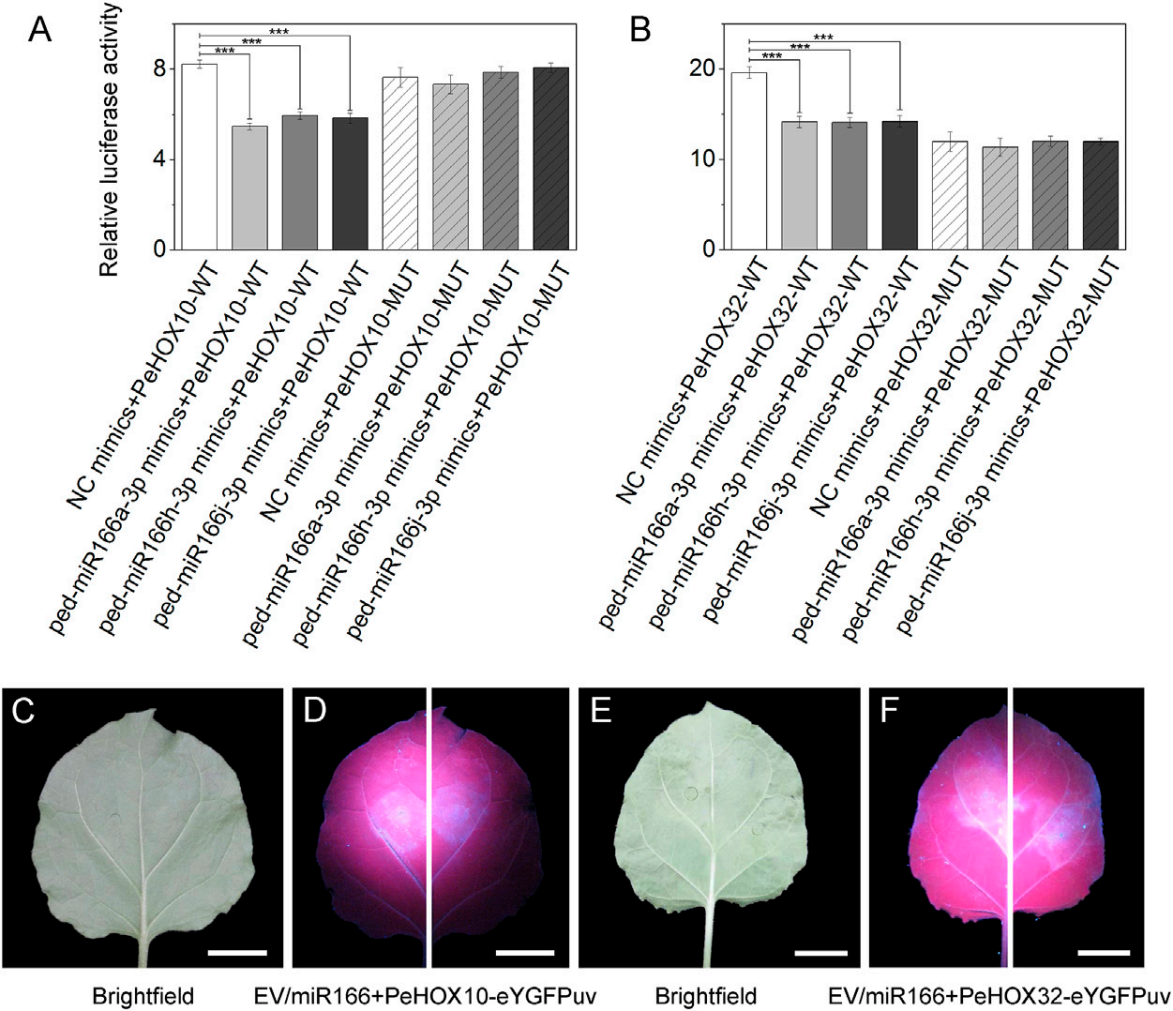
Verification of the correlation between three miR166s and two target genes using dual-luciferase reporter and transient expression assays. **(A–B)** Dual-luciferase reporter assays confirmed that miR166s are bound to *PeHOXs*. The three miRNAs were ped-miR166a-3p, ped-miR166h-3p, and ped-miR166j-3p. The two target genes were *PeHOX10* (*PH02Gene00975*) and *PeHOX32* (*PH02Gene31932*). Error bars indicate the standard deviations of three technical repeats. The significance of differences among groups was determined using *t*-tests. *p < 0.05; **p < 0.01; and ***p < 0.001. **(C–F)** Transient expression assays confirmed that miR166s are bound to *PeHOXs*. Differential fluorescence intensity was observed between two sides of the same leaf under the UV light, where an empty vector (EV)/miR166 + PeHOXs-eYGFPuv was injected. Bar = 1 cm.

Dual-luciferase reporter assays revealed that three miR166s were specifically bound to *PeHOX10* and *PeHOX32*. The luciferase activity of the miRNA mimics + mRNA-WT group was lower than that of the NC mimics + mRNA-WT group (*p* < 0.001) in the transfected cells (Figures 6A,B). However, no significant differences were observed between the two mutant groups.

Transient expression assays revealed that ped-miR166a-3p bound to *PeHOX10* and *PeHOX32*. Differential fluorescence intensity was observed on two sides of the same leaf of *N*. *benthamiana* under the UV light (Figures 6C–F). The fluorescence intensity on the left side (Empty vector + PeHOXs-eYGFPuv) of treated leaves was higher than that on the right side (miR166 + PeHOXs-eYGFPuv).

### miR166s were localized to the xylem

**FIGURE 7:**
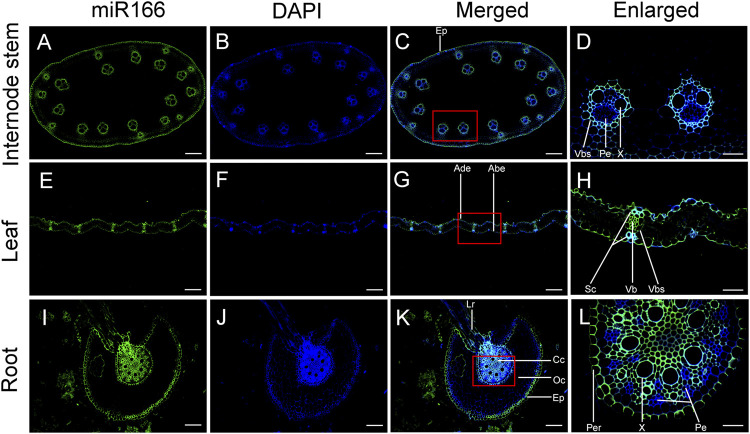
Verification of the localization of miR166s to vascular tissues of the internode, leaf, and root of two-month-old Moso bamboo seedlings by fluorescence *in situ* hybridization (FISH). ped-miR166a-3p was localized to the vascular tissues of the internode **(A–D)**, leaf **(E–H)**, and root **(I–L)**. Ep, epidermis; Vb, vascular bundle; Vbs, vascular bundle sheath; Pe, phloem; X, xylem; Ade, adaxial epidermis; Abe, abaxial epidermis; Sc, sclerenchyma; Lr, lateral root; Cc, central cylinder; Co, cortex; Per, pericycle. Bar = 100 µm.

The VBs were distributed in various tissues of bamboo seedlings, and the morphological characteristics varied among organs (Figure 7). In the roots, VBs consisted of approximately a dozen small sieve tubes surrounding seven vessels. In the stems, large VBs were located around strips and had well-developed xylem; more than ten VBs were divided into two rings. VBs in the inner ring were more developed than those in the outer ring, which were mainly distributed in the mechanical tissues. VBs were also located in the leaves and leaf sheaths, and the VB sheaths included thick-walled cells.

Differential fluorescence intensity was observed in various tissues of the internode, leaf, and root of bamboo seedlings in merged images after false-positive signals from the black controls were excluded (Supplementary Figure S1). The fluorescence intensity of miR166 was higher than that of the 4,6-diamidino-2-phenylindole 2 HCl (DAPI) stain in the epidermis, xylem, and VB sheath of the internode (Figures 7A–D). ped-miR166a-3p was localized to the adaxial epidermis, abaxial epidermis, sclerenchyma, VB, and the VB sheath of the leaf (Figures 7E–H). ped-miR166a-3p was localized to the lateral root, epidermis, cortex, xylem, and pericycle of the root (Figure 7I–L). The fluorescence intensity of miR166 was lower than that of the DAPI stain of the phloem of the internode and root. These results confirmed that ped-miR166a-3p was specifically expressed in the xylem of the internode, leaf, and root of two-month-old Moso bamboo seedlings.

## Discussion

We assessed the expression patterns of miR166s and their predicted targets and studied their interplay during tissue differentiation in Moso bamboo seedlings.

### miR166s interact with *PeHOXs* and *RLKs* during tissue differentiation

Posttranslational modifications are a major mechanism by which miRNAs repress the expression of TFs, and TFs work synergistically or antagonistically to regulate the expression of various downstream target genes. miRNAs are important parts of the miRNA–TR–plant hormone regulatory network, which regulates complex processes with distinct developmental outcomes. In Arabidopsis, HD-ZIP TFs, which are regulated by miR165/166, play multiple, possibly interdependent, roles in plant development (Byrne, 2006). In our study, HD-ZIP TFs were detected in most miR166–mRNA pairs in the regulatory network. Dual-luciferase reporter assays confirmed that miR166a/h/j bound to *PeHOX10* and *PeHOX32*, both of which are bamboo *HD-ZIP III* homologs of rice. This finding indicates that the same target could be degraded by more than one miR166. miR166s likely affect the function of the meristem by regulating the expression of *PeHOXs* and contributing to distinct developmental outcomes in the underground shoots. This also indicates that miR166s play a key role in tissue differentiation and possibly underlie the morphological innovation of the developing tissues in Moso bamboo shoots.

miR166s affected the expression of other TF genes that participate in diverse processes, including immune and stress responses, light and hormonal signaling, and development, by specifically binding to *cis*-acting elements in the promoters of their target genes. This finding indicates that the miRNA–TF–mRNA regulatory network and miR166s play key roles in tissue differentiation in Moso bamboo. PKs in the RLK/Pelle class were detected in most miRNA–mRNA pairs (13). The RLK/Pelle PKs function in various processes, such as cell wall interactions, disease resistance, and developmental control (Gish and Clark, 2011), indicating that miR166s might affect the organization of the cell wall and regulate developmental processes. miR166s also target genes encoding TRs involved in plant hormone signal transduction. For example, both ped-miR166h-3p and ped-miR166j target *PH02Gene01141*, which encodes an AUX/IAA protein. AUX/IAA family proteins mediate auxin signaling and play important roles in plant growth and development. This finding indicates that genes related to signal transduction pathways and kinase activity during tissue differentiation are particularly important in developing bamboo shoots.

### miR166s regulate the development of vascular tissues

Moso bamboo is distinguished from other monocot herbs by its tall culm, luxuriant leaves, and its complex and vast system of underground rhizomes. Strong and unimpeded vascular flow is needed to ensure that a source–sink balance is achieved. Unlike dicots, bamboo plants do not possess secondary cambia, and the multiple layers of vascular tissue in the culms provide resistance against external mechanical stress. Thus, VBs in Moso bamboo is different from and more complex than those in other dicotyledons. Li et al. (2022) found that both miR166h-3p and miR166a-3p are localized to the cambium of underground shoots at the S1 stage and in the vascular tissue of shoots at the S3 stage. This finding indicates that miR166s play an important role in vascular tissue differentiation in bamboo shoots. ped-miR166a-3p was localized to the xylems in the vascular tissue of the leaf, stem internode, and central cylinder of the root elongation zone. Therefore, ped-miR166a-3p promotes the differentiation of the vascular tissue in various organs of bamboo seedlings.

The TW Moso bamboo has a thick culm wall, with an increased number of VBs and a much narrower pith cavity compared with the WT Moso bamboo. The fact that the apical meristem of the shoot at the S1 stage in the TW Moso bamboo produces more vascular tissue as opposed to pith cells compared with the WT Moso bamboo (Wei et al., 2017) might be explained by developmental differences between WT and TW Moso bamboos. In our study, the greatest number of target DEGs regulated by miR166s was observed in the TWS2_vs._TWS1 comparison. Additionally, the expression profiles of these target DEGs varied in the WTS2_vs._WTS1 and TWS2_vs._TWS1 comparison, and this might have contributed to variation in the phenotypes between WT and TW Moso bamboos. Although only two miR166s were differentially expressed, target DEGs were detected for all seven miR166s. Five miR166s were overexpressed, and three wmiR166s were highly overexpressed. Small changes in the expression levels of these genes thus might be sufficient to upregulate and downregulate the expression of target genes.

The target DEGs in the TWS2_vs._TWS1 comparison varied among the seven miR166s. Expression patterns of DEGs and miR166s in the TWS2_vs._TWS1 comparison were inconsistent. This might be explained by the fact that miR166s and other miRNAs affected the expression of these DEGs; that is, the miR166s might specifically bind to them to control their expression at certain stages, and they might participate in different biological pathways. These findings indicate that the miRNA–mRNA regulatory network is complex. Additional analyses and experiments are needed to verify the roles of miR166s in this regulatory network. Bamboo shoots can reach 18.3 m in only 3 months (Khalil et al., 2012); the large amount of water needed for cell elongation during this process is supplied by an unobstructed transport system. The molecular regulatory network and regulatory mechanisms might become more sophisticated during the developmental process in Moso bamboo, as the rhizomes of vegetative organs mainly reproduce asexually. This also might increase the complexity of the regulatory network, given that the xylem and phloem differ in both structure and function. miR166s might play specific regulatory roles in the vascular tissues during seedling growth, especially in the xylem.

### Potential roles of miR166s in xylem patterning

miR165/6 functions as a mobile and morphogenic signal that determines the fates of cells during development. For example, miR165/6 is specifically expressed in the endodermis of the root, but a gradient of miR165/6 towards the center of the vasculature leads to an opposing gradient in the expression of its target genes (Miyashima et al., 2011; Miyashima et al., 2019). Furthermore, AGO1 preferentially recruits small RNAs with 5′-terminal uridine to construct RNA-induced silencing complexes (RISCs) to regulate gene expression at the transcriptional and posttranscriptional levels (Mi et al., 2008). Fan et al. (2022) showed that miR165/6, which is produced in the endodermis, moves to the vasculature to determine the fates of xylem cells in *Arabidopsis* roots. They also suggested that the cytoplasmic AGO1 loading of mobile miRNAs is a key step for promoting miRNA cell-to-cell movement. In this study, three overexpressed miR166s had a 5′ uridine residue as the first base, and ped-miR166a-3p was localized to the endodermis and the xylem in the roots of bamboo seedlings according to RNA FISH. These miR166s might be recruited by bamboo AGO1 to construct miRISCs that regulate the expression of *HD-ZIP* genes; they might also serve as local and long-distance signals in xylem patterning in multiple organs in Moso bamboo.

Moso bamboo shoots differ morphologically from the internodes and roots; they develop from underground vegetative buds located at the nodes of the rhizomes, which are morphologically similar to the roots but have nodes similar to the aboveground culm. In our study, ped-miR166a-3p, which has a 5′ uridine residue as the first base, was localized to the xylem rather than the phloem of the internode and the root. Li et al. (2022) detected ped-miR166a-3p in the cambium and xylem of Moso bamboo shoots at S1 and S3 stages, respectively. This indicates that miR166 is associated with xylem patterning and that there might be a relationship between the development of the shoot, root, and internode. miR166s in Moso bamboo might move between the xylem of the vasculature and function as mobile morphogenic signals that determine the fates of various types of cells during the development process. Additional experiments are needed to clarify the roles of miR166s in xylem patterning.

## Conclusion

We characterized changes in the profiles of miR166s and their predicted targets. Our findings indicate that miR166s play key roles in the differentiation of vascular tissue in Moso bamboo. The localization of ped-miR166a-3p to the xylem in the stems and roots of two-month-old seedlings indicates that it plays a key role in the development of the xylem in Moso bamboo. Only ped-miR166a-3p could be localized using RNA FISH. Consequently, its interaction with other miR166s and target genes could not be determined. Additional approaches are needed to confirm the interaction between miR166s and their predicted targets. The miR166s identified in our study could be used to improve the properties of bamboo wood and the edible quality of bamboo shoots. The findings of our study reveal a complex regulatory network, in which miR166s target *PeHOXs*, *PeNACs*, and other genes to control vascular tissue differentiation in bamboo.

## Data Availability

The original contributions presented in the study are included in the article/Supplementary Material; further inquiries can be directed to the corresponding authors.

## Supplementary material

Supplementary Figure S1 Signals of the blank control in Moso bamboo seedlings by fluorescence in situ hybridization. Bar = 20 µm expression.

Supplementary Table S1 Sequences of miR166 mimics, target gene fragments, and primers used for dual-luciferase reporter and transient expression assays.

Supplementary Table S2 Seven miR166s were identified from the small RNA sequencing data using the underground shoot samples of wild-type (WT) Moso bamboo (Phyllostachys edulis) and a thick-walled (TW) variant (P. edulis 'Pachyloen') downloaded from NCBI. Five and three miRNAs were overexpressed (TPM > 2,000) and highly overexpressed (TPM > 10,000) throughout all developmental stages in WT and TW groups, which are indicated in bold and italics, respectively. NA: not available.

Supplementary Table S3 A total of 487 predicted targets of seven miR166s. The transcription factor, protein kinase, and transcriptional regulator targets are in bold, red, and italics, respectively.

Supplementary Table S4 A total of 423 miRNA–mRNA pairs comprising five overexpressed (TPM > 2,000) miRNAs and their predicted targets. The transcription factor, protein kinase, and transcriptional regulator targets are in bold, red, and italics, respectively.

Supplementary Table S5 A total of 23 miR166–mRNA pairs were confirmed by the degradome sequencing dataset.
