# Supplementary figures and images for: The miR166–mRNA network regulates vascular tissue differentiation in Moso bamboo
Source: Front Genet. 2022 Aug 12;13:893956. doi: 10.3389/fgene.2022.893956 (PMC9412049; doi:10.3389/fgene.2022.893956)

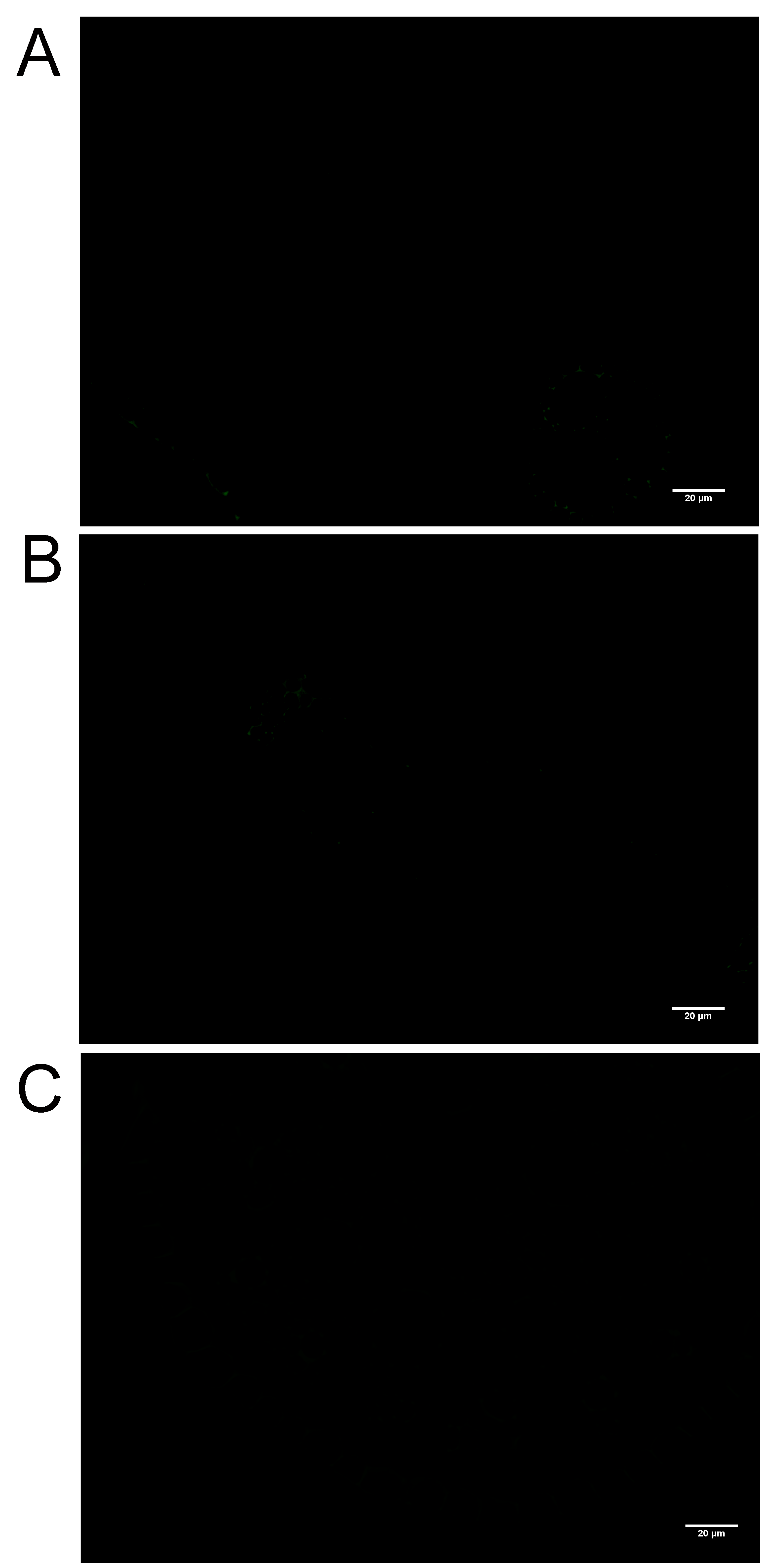

Supplement: Supplementary file 3 [file Image1.TIF]
